# Bee pollen and propolis improve neuroinflammation and dysbiosis induced by propionic acid, a short chain fatty acid in a rodent model of autism

**DOI:** 10.1186/s12944-019-1150-0

**Published:** 2019-11-16

**Authors:** Kawther Aabed, Ramesa Shafi Bhat, Abeer Al-Dbass, Nadine Moubayed, Norah Algahtani, Nada M. Merghani, Azizah Alanazi, Naima Zayed, Afaf El-Ansary

**Affiliations:** 1 Biology Department, College of Science, Princess Nourah Bint Abdulrahman University, Riyadh, Saudi Arabia; 2 Biochemistry Department, College of Sciences, King Saud University, Riyadh, Saudi Arabia; 3 Biology Department, College of Sciences, King Saud University, Riyadh, Saudi Arabia; 4 Central laboratory, Female Centre for Scientific and Medical Studies, King Saud University, Riyadh, Saudi Arabia; 5 Department of Cell Biology, King Faisal Specialist Hospital and Research Centre, Riyadh, Saudi Arabia; 6 Therapeutic Chemistry Department, National Research Centre, Dokki, Cairo, Egypt

**Keywords:** Autism, Propionic acid, Cytokines, Neuroinflammation

## Abstract

****Background**:**

Neuroinflammation plays a major role in the pathogenesis of autism because the cytokine levels are typically disturbed in the brain in autistic patients. Prebiotics-rich diet maintains the healthy gut microbiota and hence can regulate the neuroinflammation indirectly. The study aimed to investigate the role of bee pollen and propolis in ameliorating neuroinflammation, including cytokine levels, in an animal model of autism.

****Methods**:**

Hamsters were classified as four groups: Group I, control; Group II, autistic model/animals treated with 250 mg propionic acid (PPA)/kg body weight (BW)/day for 3 days; Group III, animals treated with bee pollen at a dose of 250 mg/kg BW/day for 4 weeks; and Group IV, animals treated with propolis at a dose of 250 mg/kg BW/day for 4 weeks. Neuroinflammatory responses were evaluated using the levels of interferon γ (IFN-γ), interleukin 1 alpha (IL-1α), IL-6, IL-10, IL-12 (p70), vascular endothelial growth factor (VEGF), and tumor necrosis factor α (TNFα).

****Results**:**

Significant decrease of IL-10 (*P*<0.026), VEGF (*P*<0.005), and TNFα(*P*<0.005) levels and increased IL-1α (*P*<0.032), IL-6(*P*<0.028), and IFN-γ (*P*<0.013) levels were observed between the four studied groups. The neurotoxic effects of PPA was clearly presented as much higher IL-6, as pro-inflammatory cytokine (*P*<0.05), concomitant with much lower IL-10, as anti-inflammatory cytokine(*P*<0.015) compared to controls. Both bee pollen and propolis were effective in ameliorating the neurotoxic effects of PPA demonstrating non-significant changes of IL-6 and IL-10 when compared to control healthy hamsters.

****Conclusions**:**

Our findings indicate that both bee pollen and propolis protect against neuroinflammation in the rodent model of autism. However, further studies are needed to investigate the clinical benefits of prebiotics-rich diet in neurodevelopmental disorders, such as autism.

## Background

Brain pathology in autism as a neurodevelopmental disorder is related to neuroinflammation in different brain parts. Evidence of neuroinflammation in autism includes astrocyte and microglial activation, exclusively increased proinflammatory cytokines [1].

Animal models of autism are being developed to examine the essential mechanisms, advance medicine, and identify approaches of the evaluation of symptoms of this disorder [2, 3]. Propionic acid (PPA) is an intermediate breakdown product of cellular fatty acids, and it is produced in the gut, along with other short-chain fatty acids such as acetate and butyrate, all of which are major metabolic products of enteric bacteria and are also fermentation products of nutritional carbohydrates and amino acids [4, 5]. PPA is formed by bacteria in the intestinal tract [4] and oral mucosa [6]. Because PPA is a weak organic acid and exists in ionized and non-ionized shapes at physiological pH, it easily crosses the gut-blood and blood-brain barriers and reaches the brain [7].

PPA-treated rats have typical autistic features, including hyperlocomotion, impaired social interaction, anxiety, and repetitive behavior [8, 9]. Clostridial species are the main gut colonist in primary life and are producers of PPA and other short-chain fatty acids [10]. Clostridia are resistant to most antibiotics used for frequent infections and are a reason of major hospital-acquired infectious disease [10]. Remarkably, spore-making anaerobes and microaerophilic bacteria mainly from Clostridial species have been demonstrated to be elevated in autistic patients [11, 12].

It is well accepted that immune responses can affect the functions of the endocrine and nervous systems. Immuneneuroendocrine network (INEN) is defined as the interactions between immune, nervous, and endocrine systems. The brain gets immune signals from the soluble circulating cytokines (e.g., IL-1α, IL-1β, TNF-α, and IL-6) transported through the blood-brain barrier (BBB), from peripheral immunity through the vagus nerve, and from immune cells entering the brain and the brain immune microglial cells [13, 14]. Environmental factors that disrupt INEN functions include exposures to microbial, chemical, physical, and psychological stressors. Therefore, PPA, which is a metabolic endproduct of clostridia species and is known to be 10-fold higher in individuals with autism, can be regarded as a stressor for both the immune and nervous systems [15].

Bee pollens and propolis are rich in flavonoid, trace elements, and other healthy components, and they have been demonstrated to possess several amazing health effects, such as antioxidant, anti-allergic, and anti-inflammatory properties [16]. In this study, pro-inflammatory and anti-inflammatory cytokines were investigated in PPA -treated hamsters, and the effectiveness of bee pollen and propolis (two intervention strategies) was also examined.

## Methods

### Animals

Twenty four male golden Syrian hamsters with body weight (BW) between 60 g and 100 g were used in this study, and they were bought from a live safari store in Riyadh, Saudi Arabia. Hamsters were raised strictly under a standard laboratory condition and fed on standard pellets. All the hamsters were kept individually in cage 41 (40 × 35 × 20 cm^3^), with controlled temperature (21 ± 1 °C) and light conditions (light on at 9:00 AM and light off at 21:00 PM). Hamsters had free access to food (standard laboratory animal feed pellets) and water.

### Ethics approval

The study was approved by Animal Research Committee of Princess Nourah Bint Abdulrahman University, Riyadh (Approval number, IBR- 16-0031). All the methods used in the study strictly followed the guidelines of National Institutes of Health Guide for the Care and treatment of Laboratory Animals in Experiments.

### Experimental design

**Fig. 1:**
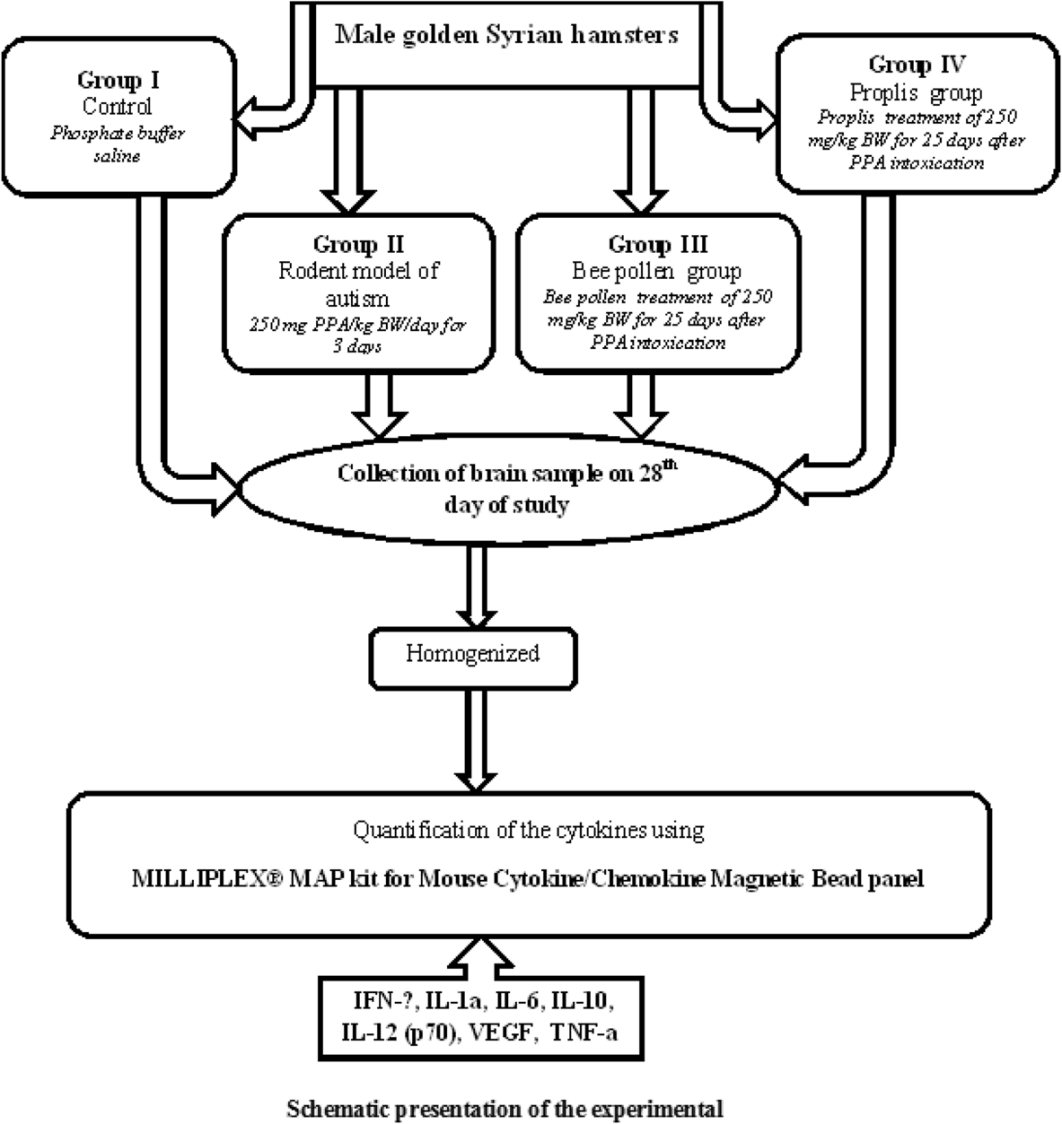
Diagrammatic presentation of the experimental work

Hamsters were randomly divided equally into four groups. Group I was assigned as control; Group II (rodent autistic model) was treated with 250 mg PPA/kg BW/day for 3 days; Group III was fed with bee pollen at a dose of 250 mg/kg BW for 25 days after PPA intoxication; Group IV was fed with propolis at the dose of 250 mg/kg BW for 25 days after PPA intoxication. A descriptive diagram for the experimental work is presented in Fig. 1.

### Preparation of brain homogenates for identification of cytokines

At the end of the study, which lasted for 4 weeks, all the animals were sacrificed, and the whole brain was collected. The brain tissue was homogenized with Tissue Lyser LT (QIAGEN) at a high-speed of 50 rotations per minute, and the homogenate was then centrifuged at 4000 rpm for 15 min before the identification of the specific cytokines.

### Quantification of cytokines in the brain tissue

MILLIPLEX® MAP kit for Mouse Cytokine/Chemokine Magnetic Bead panel was used for quantification of the cytokines in the brain homogenate according to the manufacturer’s instruction. A panel of measured cytokines comprised interferon γ (IFN-γ), interleukin 1 alpha (IL-1α), IL-6, IL-10, IL-12 (p70), vascular endothelial growth factor (VEGF), and tumor necrosis factor α (TNF-α).

### Identification of *C. difficile* in fecal samples using a toxin assay

All the fecal samples except those from the bee pollen group were preliminary positive for *Clostridium difficile* growth, manifesting as yellow colonies with glass texture on CCFA agar plates. *C. difficile* Quick check (TECHLAB, Alere, USA) was performed according to the manufacturer’s instruction for further identification of *C. difficile* in the positive groups.

### Statistical analyses

The data in the present study are expressed as means ± standard deviation (SD). All statistical comparisons between the control hamster group and the PPA-, bee pollen-, and propolis-treated hamster groups were performed using one-way analysis of variance (ANOVA) tests with Dunnett’s test for multiple comparisons. Statistical Package for the Social Sciences (SPSS, Chicago, IL, USA) was used. Significance was assigned at a *P* level of < 0.05. Receiver operating characteristics curve (ROC) analysis was performed. The area under the curve (AUC), cutoff values, and the degrees of specificity and sensitivity were calculated. Pearson’s correlations were performed between the measured parameters. Multiple regression analysis was also performed between the different measured variables.

## Results

Significantly lower levels of IL-10 (64.11% decrease), VEGF (9.9%), and TNFα (15.22%) together with increased IL-1α (20.33%), IL-6 (327.03%), and IFN-γ (5.29%) levels were observed in PPA-treated group compared to controls as shown in Table 1. The neurotoxic effects of PPA was clearly presented as much higher IL-6, as pro-inflammatory cytokine (P<0.05), together with much lower IL-10, as anti-inflammatory cytokine(P<0.015) compared to controls. Both bee pollen and propolis were effective in ameliorating the neurotoxic effects of PPA demonstrating non-significant changes of IL-6 and IL-10 when compared to control healthy hamsters (Table 1). Table 2 shows the multiple regression analysis of the measured cytokines with IL-12 (P70) (pg/ml) and TNFα (pg/ml) as dependent variables respectively. The influence of TNFα, VEGF, and IL-1α as independent variables on IL-12 (P70) as dependent variable can be easily observed from the adjusted R^2^. While TNFα alone influences IL-12 with 52.6%, there were a remarkable increase in the influence when combined with VEGF (57.7%), and with both VEGF, and IL-1α (63.1%).

**Table 1:** Mean ± S. D of the measured variables in the four studied groups

Parameters	Groups	Mean ± S.D.	*P* value^**a**^	P value^**b**^
IFNG (pg/ml)	Control	120.70 ± 167.85		0.046
	PPA	127.08 ± 192.57	1.000	
	Be pollen	17.05 ± 26.48	0.024	
	Propolis	23.23 ± 22.52	0.337	
IL-1A (pg/ml)	Control	181.05 ± 65.52		0.035
	PPA	217.85 ± 76.89	0.531	
	Be pollen	116.39 ± 40.72	0.109	
	Propolis	168.96 ± 15.29	0.981	
IL-6 (pg/ml)	Control	18.87 ± 4.69		0.091
	PPA	80.56 ± 63.58	0.050	
	Be pollen	21.94 ± 25.93	0.336	
	Propolis	21.58 ± 15.36	0.630	
IL-10 (PG/ML)	Control	213.86 ± 212.98		0.050
	PPA	76.76 ± 36.44	0.015	
	Be pollen	94.08 ± 28.87	0.022	
	Propolis	231.39 ± 270.26	0.572	
IL-12(P70) (pg/ml)	Control	82.07 ± 8.65		0.094
	PPA	71.03 ± 7.57	0.336	
	Be pollen	70.11 ± 13.10	0.271	
	Propolis	69.14 ± 7.35	0.214	
VEGF (pg/ml)	Control	5.95 ± 2.43		0.010
	PPA	5.36 ± 2.64	0.575	
	Be pollen	3.58 ± 0.85	0.025	
	Propolis	3.41 ± 0.53	0.010	
TNFA (pg/ml) ^**c**^	Control	17.11 ± 1.39		0.003
	PPA	14.50 ± 0.91	0.140	
	Be pollen	13.95 ± 3.27	0.057	
	Propolis	11.72 ± 2.24	0.001	

^**a**^ *P* value between each group and the control group

^**b**^ *P* value among all groups

**Table 2:** Multiple regression analyses using a stepwise method with IL-12 (P70) (pg/ml) and TNFα (pg/ml) as dependent variables

Dependent variables	Predictor variables	Coefficients	*P* values	Adjusted R^2^	95% CIs	
					Lower	Upper
IL-12 (P70) (pg/ml)	TNFα	3.454	0.000	0.526	2.227	4.681
	TNFα	3.873	0.000	0.577	2.641	5.106
	VEGF	−1.755	0.047		−3.480	−0.029
	TNFα	3.874	0.000	0.631	2.720	5.027
	VEGF	−2.143	0.013		−3.798	−0.489
	IL-1A	0.056	0.035		0.004	0.108
TNFα (pg/ml)	IL-12 (P70) (pg/ml)	0.157	0.000	0.526	0.101	0.213
	IL-12 (P70) (pg/ml)	0.157	0.000	0.624	0.107	0.206
	VEGF (pg/ml)	0.459	0.008		0.132	0.786

IL-12, interleukin-12; TNFα, tumor necrosis factor α; CIs, *confidence interval*s; VEGF, vascular endothelial growth factor

Additionally, IL-12 as predictor variable influences TNFα as dependent variable with 52.6%, and much higher influence was reported for IL-12 and NEGF together as predictor variables (62.4%). Table 3 presents the ROC analysis of the measured cytokines in the four studied groups. It can be easily noticed that among the measured cytokines, ILs-6, − 10, and − 12 together with TNFα recorded the much higher AUCs as predictive markers of PPA neurotoxicity, and therapeutic effects of bee pollen and propolis. Table 4 shows the presence or absence of *C. difficile* antigen, toxin A, and toxin B in the four studied groups. *C. difficile* antigen was present in three studied groups, and toxins A and B were not detected in the bee pollen group.

**Table 3:** ROC-Curve of various parameters in all groups

Parameters	Groups	AUC	Cut-off value	Sensitivity %	Specificity %	P value	95% CI
IFN-γ (pg/ml)	PPA	0.500	25.380	50.0%	66.7%	1.000	0.153–0.847
	Bee pollen	0.889	8.445	83.3%	100.0%	0.025	0.673–1.105
	Propolis	0.667	17.380	83.3%	66.7%	0.337	0.331–1.002
IL-1A (pg/ml)	PPA	0.694	172.545	66.7%	66.7%	0.262	0.385–1.004
	Bee pollen	0.861	129.458	66.7%	100.0%	0.037	0.645–1.077
	Propolis	0.569	152.360	83.3%	50.0%	0.689	0.210–0.928
IL-6 (pg/ml)	PPA	0.833	23.195	83.3%	83.3%	0.055	0.570–1.096
	Bee pollen	0.667	16.880	66.7%	83.3%	0.337	0.317–1.017
	Propolis	0.583	18.145	66.7%	66.7%	0.631	0.241–0.926
IL-10 (PG/ML)	PPA	0.917	122.270	100.0%	83.3%	0.016	0.742–1.091
	Bee pollen	0.889	119.215	100.0%	83.3%	0.025	0.673–1.105
	Propolis	0.597	122.055	66.7%	83.3%	0.575	0.239–0.956
IL-12 (P70) (pg/ml)	PPA	0.833	72.835	66.7%	100.0%	0.055	0.593–1.073
	Bee pollen	0.861	72.130	83.3%	100.0%	0.037	0.604–1.119
	Propolis	0.903	72.130	83.3%	100.0%	0.020	0.708–1.098
VEGF (pg/ml)	PPA	0.597	4.500	66.7%	66.7%	0.575	0.253–0.941
	Bee pollen	0.889	4.240	83.3%	83.3%	0.025	0.697–1.081
	Propolis	0.944	3.645	83.3%	100.0%	0.010	0.814–1.075
TNFα (pg/ml)	PPA	0.944	15.095	83.3%	100.0%	0.010	0.814–1.075
	Bee pollen	0.833	14.845	83.3%	100.0%	0.055	0.535–1.132
	Propolis	1.000	14.680	100.0%	100.0%	0.004	1.000–1.000

ROC, receiver operating characteristics curve; AUC, area under the curve; IFN-γ, interferon γ

**Table 4:** Identification of *C. difficille by a* toxin assay

Fecal samples	Antigen	Toxin A	Toxin B	*C. difficile*
PPA	**+**	**+**	**+**	**+**
Bee pollen	**–**	**–**	**–**	**–**
Propolis	**+**	**–**	**–**	**+**
*C. difficile*	**+**			**+**

Antigen, glutamate dehydrogenase antigen; PPA, propionic acid; **+**, positive; −, negative

If Antigen is positive and toxins A and B are negative, non-toxin-producing *C. difficile* is present

If Antigen, toxin A, and toxin B are positive, toxin-producing *C. difficile* is present

## Discussion

**Fig. 2:**
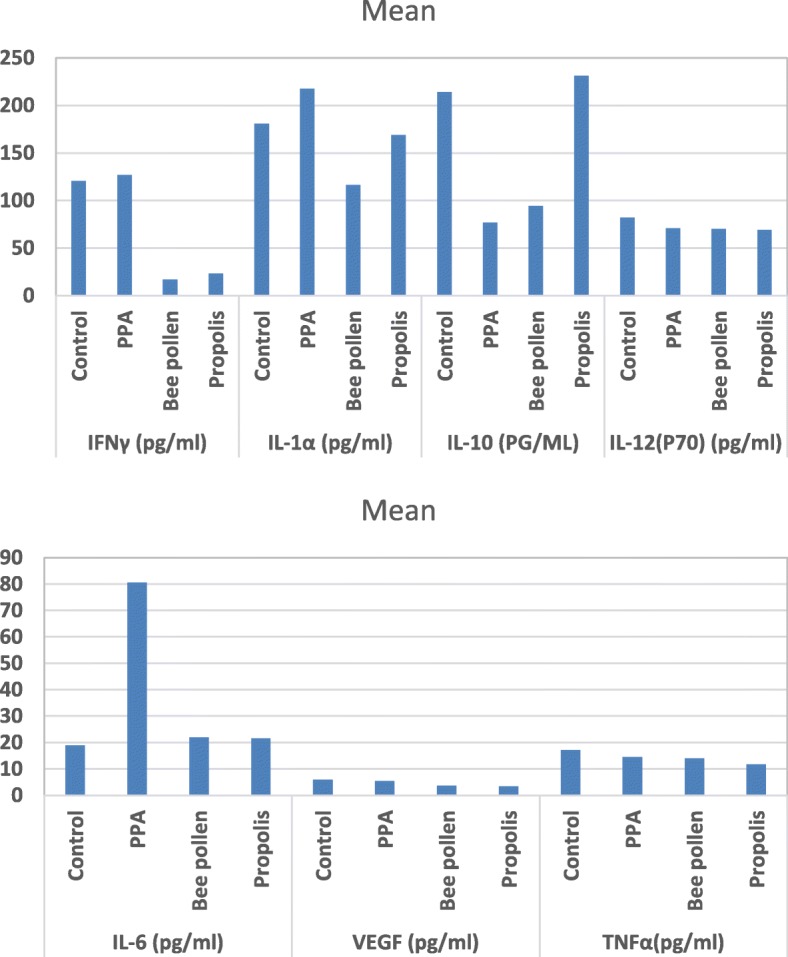
Mean of the measured cytokines in the four studied groups

The present study demonstrates the immune variation of the neurotoxic properties of PPA and the remarkable ameliorating effects of bee pollen and propolis as prebiotics because they can induce the growth of healthy bacteria and reduce the overgrown of pathogenic *C. difficile* (Table 1 and Fig. 2).

This can find support in multiple studies which have investigated the biochemical, physiological, pharmaceutical, and medicinal properties of bee pollen and propolis as poly-phenolic and flavonoids –rich products. It is well known that both demonstrate free radical scavenging activity and multiple pharmaceutical potencies, including anti-inflammatory, immune-modulatory, and antioxidant activity [16].

IL-1 is a pro-inflammatory cytokine and a key mediator in neuronal toxicity and death. Treatment of purified astrocytes or co-cultures of neurons and glial cells with IL-1 usually induces caspase activation and results in neuronal death. Neuronal cell death induced by IL-1 is prevented by pre-treatment with the IL-1 receptor antagonist, caspase inhibitor, or the antioxidant α-tocopherol, a component of bee pollen and propolis [16]. The IL-1 expression level dramatically increases in the brain after acute brain insults in rodents [17]. Moreover, it is well known that multiple neuro-inflammatory mediators, which are involved in the etiology of autism, are usually released in response to IL-1 α; these mediators include IL-6, TNF- α, prostaglandins, and cyclooxygenase-2 [18, 19]. Therefore, the 20% increase of IL-1α in PPA-treated hamsters (Table 1 and Fig. 2) may indicate the neurotoxic effects of orally administered PPA [8]. The neurotoxic effects are also related to the dramatic increase of IL-6 (a 327.03% higher level) in PPA-treated animals (P < 0.05).

Multiple studies have demonstrated that IL-6 is functionally linked to the acute phase inflammatory C-reactive protein (CRP). During the elevation of IL-6, the pro-inflammatory cytokines, including IL-1 and TNF-α, promote the expression of CRP. The neurotoxic effects of PPA, manifesting as the remarkable increase in IL-1α and IL-6, were clearly observed in this study (Table 1). Notably, both IL-6 and CRP levels are elevated in the plasma in the individuals with autism. The findings are supported by those in a recent study by Sharma, in which PPA-treated animals showed enhanced inflammation, manifesting as increased IL-6 and TNF-α and decreased interleukin-10, in different brain regions [9].

**Fig. 3:**
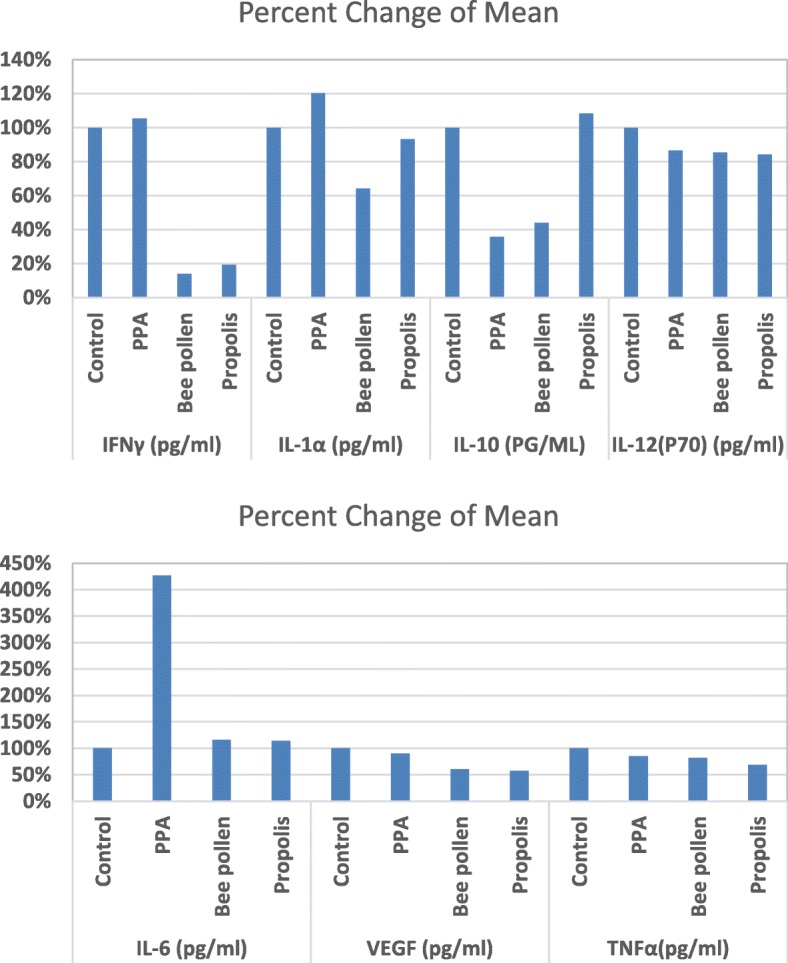
Percentage change of all parameters in all groups compared to control

Interleukin-10, an anti-inflammatory cytokine, exerts a surplus of immunomodulatory functions during an inflammatory response. It is initially defined as a proinflammatory cytokine synthesis inhibitory factor, retards inflammation by reducing cytokine receptor expression and inhibiting receptor activation [20]. Therefore, the significant decrease in IL-10 and the significant increase in IL-6 clearly suggest the neurotoxic effects of PPA (Table 1 and Figs. 2 and 3).

Prebiotics, such as dietary fibers and some oligosaccharides, have been shown to have beneficial effects on human health [21]. It has been widely demonstrated that ingestion of prebiotics is associated with changes in gut microbes. For example, a 10-fold increase in fecal Bifidobacteria has been demonstrated in participants receiving oligosaccharides compared with those receiving placebos [22]. Recently, bee pollen from Saudi Arabian origin has been found to be rich in oligosaccharides, which can induce a remarkable reduction in the Clostridia species [23, 24].

Bee therapy or apitherapy is the use of honey bee products for therapeutic purposes. Bee pollen and propolis are among the medicinal products of the bee. The neuroinflammation process involves the release of cytokines (immune mediators) from neurons or damaged tissues. Table 1 shows the therapeutic effect of bee pollen and propolis, and it can be easily noticed that bee pollen and propolis have anti-inflammatory effects, manifesting as remarkable decreases in IFN-γ, IL-1α, Il-6, VEGF, and TNF-α and a significant increase in IL-10, an anti-inflammatory cytokine. These findings are consistent with those in one previous study by Hegazi [25], which indicates that bee pollen and propolis contain active substances in the floral origin of honey bee and plants, and these active substances can act upon both innate and adaptive immune responses. These compounds decrease pro-inflammatory cytokine synthesis and superoxide anion production in rabbit neutrophils. However, the activity of bee pollen is relatively lower than that of propolis, especially in terms of the induction of IL-10, an anti-inflammatory cytokine; this might be because of the higher amount of polyphenols, which are anti-oxidant and anti-inflammatory compounds, in propolis than in bee pollen [26].

Angiogenesis, an accepted mechanism in gut-microbiota related inflammation, is critically required for mucosal remodeling during treatment [27]. Therefore, the significant decrease in VEGF in bee pollen and propolis-treated groups suggests that the therapeutic effects of both might be mostly through the gut-brain axis. Neutralization of pathogenic bacterial overgrowth and partial correction of the impaired intestinal host-pathogen interaction (Table 4) can ameliorate the neurotoxic effect of PPA (impaired cytokines) [28].

As demonstrated by DeGrandi et al. between [29], the present study indicates that bee pollen is enriched in bactericidal combinations, as well as carbohydrates and lactic acid, and these ingredients are potent in reducing the growth of microorganisms, among which microbial species, including *C. difficile*, *Klebsiella pneumonia* types, and the *Candida albicans,* are known to be high in autistic patients compared with control healthy individuals [10–12].

Both glutamate excitotoxicity and neuroinflammation are well-known etiological mechanisms of several neurodevelopmental disorders, including autism, and it is suggested that decreased glutamate uptake by astrocytes could result from local inflammation [30]. Multiple studies have demonstrated that increases in IL-6 and IL-1 and a decrease in IL-10 are involved in the cellular mechanisms underlying upregulation of excitatory glutamatergic transmission and downregulation of inhibitory GABAergic transmission [31, 32]. Therefore, the remarkable alterations of these cytokines in response to PPA treatment and the significant amelioration of the alterations observed after bee pollen and propolis treatments highlight the importance of neuroinflammation and glutamate excitotoxicity as two inter-related signaling pathways, which should be targeted as therapeutic strategies of PPA neurotoxicity in rodent models of autism. The speculation can be supported by the findings in our recent study, in which bee pollen was effective in ameliorating the glutamate excitotoxicity and the impaired glutamine-glutamate-GABA circuit, two etiological mechanisms in PPA-induced neurotoxicity.

The reported pollen’s anti-inflammatory effect could be easily attributed to the presence of flavonoids, fatty acids, and phytosterols. Pollen is also rich with quercetin, which inhibits the cascade of arachidonic acid metabolism, decreasing the level of pro-inflammatory prostaglandins, having an anti-inflammatory effect [33]. On the other, hand, propolis is rich in cinnamic acid, p-coumaric acid, caffeic acid, ferulic acid, as phenolic compounds, and monoterpenes and sesquiterpenes as terpenoids with potent anti-inflammatory and antinociceptive activities [34].

## Conclusions

The present study ascertains the neuro-inflammatory effects of PPA, and the findings suggest that bee pollen and propolis supplementation reduces the inflammatory response and endotoxemia by ameliorating dysbiosis in hamsters. However further studies on the mechanisms involved in apitherapeutic intervention should be encouraged.

## Data Availability

Raw data can be available on request.

## Abbreviations

AUC: Area under the curv
BBB: Blood-brain barrier
BW: Body weight
CRP: C-reactive protein
GP130: Glycoprotein 130
IFN-γ: Interferon γ
INEN: Immuneneuroendocrine network
PPA: Propionic acid
STAT3: Signal transducer and activator, transcription 3
TNFα: Tumor necrosis factor α
VEGF: Vascular endothelial growth factor
