# Correction to: Bee pollen and propolis improve neuroinflammation and dysbiosis induced by propionic acid, a short chain fatty acid in a rodent model of autism

**DOI:** 10.1186/s12944-021-01484-y

**Published:** 2021-07-04

**Authors:** Kawther Aabed, Ramesa Shafi Bhat, Abeer Al-Dbass, Nadine Moubayed, Norah Algahtani, Nada M. Merghani, Azizah Alanazi, Naima Zayed, Afaf El-Ansary

**Affiliations:** 1 Biology Department, College of Science, Princess Nourah Bint AbdulrahmanUniversity, Riyadh, Saudi Arabia; 2 Biochemistry Department, College ofSciences, King Saud University, Riyadh, Saudi Arabia; 3 Biology Department,College of Sciences, King Saud University, Riyadh, Saudi Arabia; 4 Centrallaboratory, Female Centre for Scientific and Medical Studies, King SaudUniversity, Riyadh, Saudi Arabia; 5 Department of Cell Biology, King FaisalSpecialist Hospital and Research Centre, Riyadh, Saudi Arabia; 6 TherapeuticChemistry Department, National Research Centre, Dokki, Cairo, Egypt

**Correction to: Lipids Health Dis 18, 200 (2019)**

**https://doi.org/10.1186/s12944-019-1150-0**

Following publication of the original article [1], it was noticed that the published version of this article has typographical error in the Acknowledgements and Funding Declarations. Specifically, the “Princess Nora Bint Abdulrahman University” should be “Princess Nourah Bint Abdulrahman University”. The original article has been corrected.
